# Enhanced Reversible Zinc Ion Intercalation in Deficient Ammonium Vanadate for High-Performance Aqueous Zinc-Ion Battery

**DOI:** 10.1007/s40820-021-00641-3

**Published:** 2021-04-30

**Authors:** Quan Zong, Wei Du, Chaofeng Liu, Hui Yang, Qilong Zhang, Zheng Zhou, Muhammad Atif, Mohamad Alsalhi, Guozhong Cao

**Affiliations:** 1 Department of Materials Science and Engineering, University of Washington, Seattle, WA 98195 USA; 2 School of Materials Science and Engineering, State Key Lab Silicon Mat, Zhejiang University, Hangzhou, 310027 People’s Republic of China; 3 Research Chair On Laser Diagnosis of Cancers, Department of Physics and Astronomy, College of Science, King Saud University, Riyadh, 11451 Saudi Arabia; 4 Department of Physics and Astronomy, College of Science, King Saud University, Riyadh, 11451 Saudi Arabia

**Keywords:** Deficient ammonium vanadate, Large interlayer spacing, Reversible redox reaction, Electrochemical mechanism

## Abstract

Ammonium vanadate with bronze structure (NH_4_V_4_O_10_) is a promising cathode material for zinc-ion batteries due to its high specific capacity and low cost. However, the extraction of ${\text{NH}}_{{4}}^{ + }$ at a high voltage during charge/discharge processes leads to irreversible reaction and structure degradation. In this work, partial ${\text{NH}}_{{4}}^{ + }$ ions were pre-removed from NH_4_V_4_O_10_ through heat treatment; NH_4_V_4_O_10_ nanosheets were directly grown on carbon cloth through hydrothermal method. Deficient NH_4_V_4_O_10_ (denoted as NVO), with enlarged interlayer spacing, facilitated fast zinc ions transport and high storage capacity and ensured the highly reversible electrochemical reaction and the good stability of layered structure. The NVO nanosheets delivered a high specific capacity of 457 mAh g^−1^ at a current density of 100 mA g^−1^ and a capacity retention of 81% over 1000 cycles at 2 A g^−1^. The initial Coulombic efficiency of NVO could reach up to 97% compared to 85% of NH_4_V_4_O_10_ and maintain almost 100% during cycling, indicating the high reaction reversibility in NVO electrode.

**Supplementary Information:**

The online version contains supplementary material available at 10.1007/s40820-021-00641-3.

## Highlights

- The partial removal of ammonium cations from ammonium vanadate results in an expanded interplanar space.
- The deficient ammonium vanadate exhibits highly reversible redox reaction.
- Ex situ characterizations suggest the reversible Zn_3_V_2_O_7_(OH)_2_·2H_2_O formation/decomposition in deficient ammonium vanadate during charge/discharge processes.

**Supplementary Information:**

The online version contains supplementary material available at 10.1007/s40820-021-00641-3.

## Graphical abstract

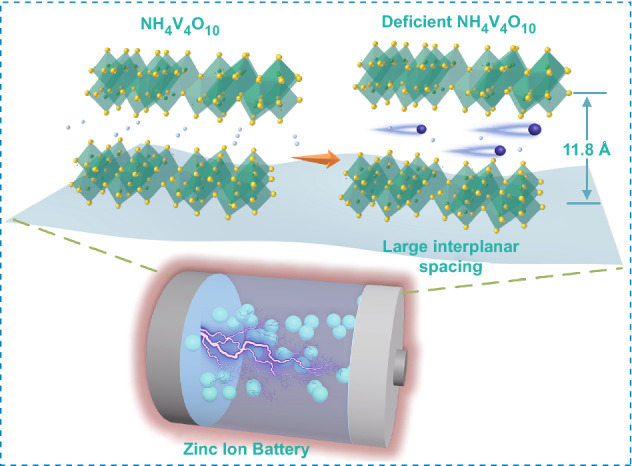

## Introduction

The global energy crisis and environmental issues continuously drive the development of renewable energy sources such as solar, wind and tidal energy [1, 2]. Electrical energy storage (EES) systems are vital to enable and guarantee the reliability and scalability of these renewable energies [3, 4]. Among various EES systems, lithium-ion batteries (LIBs) have been widely employed in the smart electronics and electric vehicles owing to their high energy density and the long-term lifespan [5–7]. Nevertheless, the safety issues, high cost, environmental concerns, as well as limited lithium resources have strongly restricted their large-scale and sustainable applications [8, 9]. Recently, a variety of aqueous batteries based on alkali metal cations (Li^+^, Na^+^, and K^+^) and multivalent charge carriers (Mg^2+^, Ca^2+^, Zn^2+^, and Al^3+^) have attracted great attention [10–14]. Aqueous zinc-ion batteries (ZIBs) are increasingly developed owing to their low cost, excellent safety, relatively high theoretical capacity (820 mAh g^−1^) and low redox potential (− 0.76 V *vs.* the standard hydrogen electrode) [15–17]. To date, a lot of attention has been paid on the cathode materials for ZIBs including manganese oxides [18–20], vanadium-based compounds [21–25], Prussian blue and its analogs [26], transition metal dichalcogenides [27, 28], and polymers [29]. However, some disadvantages seriously hinder the practical application of these materials, such as irreversible phase transition of manganese oxides, low specific capacity of Prussian blue and its analogs (< 100 mAh g^−1^), and low electrical conductivity of transition metal dichalcogenides [30–32].

Layered vanadate-based compounds have become the most competitive cathode materials for ZIBs owing to the multivalence state of vanadium ions from 5 + to 3 + and tunable layer structure with sufficient active sites for Zn-ion transport and accommodation, such as V_2_O_5_ and VO_2_ [33–35]. However, V_2_O_5_ suffers from the sluggish kinetics due to the narrow interplanar spacing (4.4 Å) and poor electrical conductivity (10^−2^–10^−3^ S cm^−1^) [36, 37]. The interplanar spacing of layered materials is critical to the electrochemical properties of electrodes [38–40]. To modify the reaction kinetics of V_2_O_5_, ions or molecules have been introduced into the interlayers, such as Na^+^ [41], K^+^ [42], Cu^2+^ [43], H_2_O [44], and PANI [45]. These ions or molecules acting as “pillars” not only accelerate zinc ions insertion/extraction but also stabilize the layered host structure. Recently, layered ammonium vanadate has attracted great attention due to the relatively large ionic size of ${\text{NH}}_{{4}}^{ + }$ in the layers and small molecular weight, providing high specific capacity [46, 47]. Various ammonium vanadates have been studied as cathode materials for ZIBs, including (NH_4_)_2_V_6_O_16_·1.5H_2_O [48], (NH_4_)_0.5_V_2_O_5_ [49], NH_4_V_4_O_10_ [50], and (NH_4_)_2_V_4_O_9_ [51]. Tang et al. investigated the electrochemical performances of NH_4_V_4_O_10_, NH_4_V_3_O_8_, and (NH_4_)_2_V_3_O_8_ with different interplanar spacing as the cathodes for ZIBs [52]. Among these ammonium vanadates, NH_4_V_4_O_10_ with the largest interplanar spacing of 9.8 Å shows the best electrochemical performance due to abundant accessible sites for reversible Zn^2+^ intercalation/deintercalation. The results demonstrate that the interplanar spacing and vanadium in multiple oxidation states play an important role on the performance improvements of ammonium vanadates cathode materials. However, ammonium vanadates often suffer from structural degradation due to the irreversible extraction of ammonium ions during the cycling test [53, 54]. These ammonium ions either enter the electrolyte to affect the PH which could degrade electrochemical properties or release toxic ammonia gas which possibly causes the safety problems. And too many ammonium ions in the interlayer could restrict the insertion of zinc ions due to the large electrostatic interactions. The impacts of pre-removing ammonium ions from the ammonium vanadates have not been explored in the ZIBs yet.

In this work, we prepared 2D NVO nanosheets through heat treatment of NH_4_V_4_O_10_ nanosheets grown on carbon cloth in air at a low temperature (300 ℃). After the heat treatment, the interplanar spacing is enlarged and the ions transfer pathways are increased due to the loss of most ${\text{NH}}_{{4}}^{ + }$ ions, which is beneficial for rapid Zn-ion intercalation/deintercalation. The layered structure of NVO remains to be stable and the NVO electrode exhibits high reaction reversibility because the deammoniation from the interplanar spacing is effectively prevented, contributing to a long-life span. The electrochemical mechanism in NVO, involving the process of ions intercalation/deintercalation, has been elaborated.

## Experimental

### Synthesis of NVO Nanosheets

All chemical reagents were used without any further purification. Prior to the synthesis, a piece of carbon cloth (1 × 2 cm^2^) was treated by sonication in 3 M HCl solution for 30 min, followed by sonication in acetone, deionized (DI) water, and absolute ethanol sequentially for 30 min each, and dried at 60 °C under vacuum for 12 h. The NH_4_V_4_O_10_ nanosheets were fabricated through a facile hydrothermal reaction. 4 mmol of NH_4_VO_3_ (98%, Fisher Scientific) and 4.8 mmol of H_2_C_2_O_4_·2H_2_O (98%, Fisher Scientific) were added into 80 mL DI water and kept stirring for 30 min. The mixture was transferred to a 100 mL-Teflon-lined stainless-steel autoclave, and then a piece of carbon cloth protected by Polytetrafluoroethylene tape was immersed into the reaction solution. The autoclave was sealed and maintained at 180 °C for 6 h. After cooling to ambient temperature naturally, the carbon cloth was carefully taken out and rinsed by ethanol and deionized water several times and then dried at 60 °C under vacuum for 12 h. The NVO nanosheets were obtained by annealing NH_4_V_4_O_10_ nanosheets at 300 ℃ in air for 2 h with the heating speed of 5 ℃ min^−1^. For comparison, the NH_4_V_4_O_10_ nanosheets were annealed at 400 ℃ in air for 2 h with the same heating speed to prepare V_2_O_5_ nanosheets. The mass load of NH_4_V_4_O_10_, NVO and V_2_O_5_ nanosheets was about 0.7, 0.65 and 0.5 mg cm^−2^, respectively.

### Material Characterizations

The phase of the sample was identified by a Bruker X-ray diffractometer (XRD, D8 Discover with IμS 2-D detection system) at an accelerating voltage of 50 kV and a working current of 1000 μA. Thermogravimetric analysis (TGA) was conducted using a TGA5500 to analyze the thermal stability of the sample within 30–600 °C. Raman spectra were recorded on a Horiba Scientific LabRAM HR Evolution with a laser excitation sources of 514 nm. Fourier transform infrared spectroscopy (FTIR) pattern was collected on Thermo Fisher Nicolet iS5 from 4000 to 400 cm^−1^ using the ATR technique. The morphologies and structures of the materials were observed using scanning electron microscopy (SEM, SU-8010) and transmission electron microscopy (TEM, Tecnai G2 F20) equipped with an energy-dispersive X-ray spectrometer (EDS) operated at 200 kV. X-ray photoelectron spectroscopy (XPS) technique was carried out on Thermo Scientific K-Alpha with an Al Kα radiation (1486.6 eV) to determine the valent state of elements.

### Electrochemical Characterizations

Electrochemical performance was tested using CR2032 coin-type cells, which were assembled by binder-free nanosheets as the cathode, Zn metal as the anode (0.15–0.25 mm thick), 80 μL of 3 M zinc trifluoromethanesulfonate (98%, Zn(CF_3_SO_3_)_2_) aqueous solution as electrolyte and a glass fiber filter (Whatman, Grade GF/A) as the separator in an air atmosphere. Cyclic voltammetry (CV) in a voltage window of 0.2–1.6 V and electrochemical impedance spectroscopy (EIS) in the frequency range from 0.01 Hz to 100 kHz were tested on a Solartron electrochemical station (SI 1287) coupled with an electrochemical impedance spectroscopy system (EIS, SI 1260). The galvanostatic charge–discharge was obtained on a Neware tester (CT-4008). The galvanostatic intermittent titration technique (GITT) was applied to analyze the reaction and diffusion kinetics at a current density of 50 mA g^−1^ and a charge/discharge time and interval of 10 min for each step.

## Results and Discussion

### Phase and Structural Characterizations

**Fig. 1:**
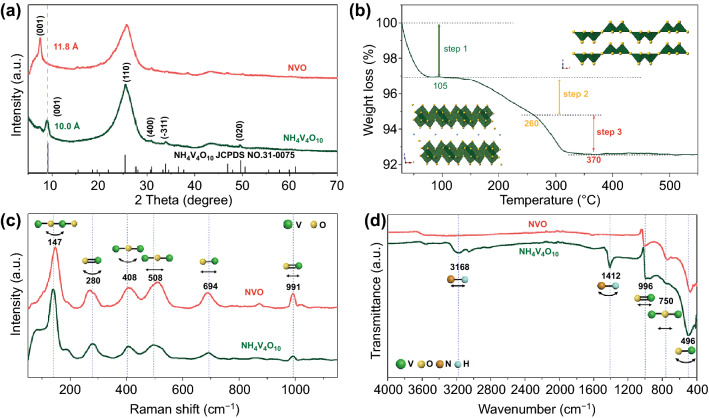
Phase and structural characterization of NH_4_V_4_O_10_ and NVO. **a** XRD patterns. The (001) peak in NVO shifts toward a low angle, indicating a large interlayer spacing. **b** TGA analysis of NH_4_V_4_O_10_. The inset is the crystal structures of bi-layered NH_4_V_4_O_10_ and single layered V_2_O_5_. **c** Raman and **d** FTIR spectra, the similar spectra indicate similar VO framework in two samples while the peak changes originate from the loss of ${\text{NH}}_{{4}}^{ + }$

XRD patterns of NH_4_V_4_O_10_ and NVO are compared in Fig. 1a, NH_4_V_4_O_10_ is well indexed to the standard pattern (JCPDS No. 31–0075), belonging to space group of C2/m with the lattice parameters, a = 11.568 Å, b = 3.652 Å, c = 9.815 Å, and β = 100.09°. The strong (001) peak located at 8.84° corresponds to a lattice spacing of 10.0 Å, which is similar to the reported 9.8 Å [54]. After annealing at 300 °C in an air atmosphere, the (001) peak in NVO moving to 7.46° suggests a large lattice spacing of 11.8 Å. In addition, new weak peaks assigned to V_2_O_5_ can be observed in this sample as shown in the enlarged patterns, which can be attributed to uneven heat transfer (Fig. S1). The little V_2_O_5_ almost has no influence on the electrochemical properties of NVO and it will be discussed later. After further annealing in air at 400 °C, the sample shows good crystallinity of orthorhombic α-V_2_O_5_ with the (001) interlayer spacing decreasing to 4.4 Å (Fig. S1). This process can be further confirmed according to TGA curve as shown in Fig. 1b. The weight loss in the first step (before ~ 105 °C) and second step (105 ~ 260 °C) originate from the removal of physically absorbed water and crystal water, respectively. The formula of the NH_4_V_4_O_10_ sample with crystal water should be NH_4_V_4_O_10_·0.49H_2_O. In the third step, the weight loss after ~ 260 °C is caused by the release of NH_3_. The NVO sample acquired via heat treatment at 300 °C has lost many ${\text{NH}}_{{4}}^{ + }$ ions from TG analysis. From the TG results, the formula of the NVO sample can be calculated as (NH_4_)_0.55_V_4_O_10_. The expanded interplanar spacing in NVO is probably attributed to the weakened interaction of ${\text{NH}}_{{4}}^{ + }$ with oxygen anion and the enlarged repelling forces between oxygen anions in the layers which could be analyzed from the crystal structure in Fig. 1b. The monoclinic NH_4_V_4_O_10_ consists of distorted VO_6_ octahedra shared edges, forming a stable bi-layered structure with the inserted ${\text{NH}}_{{4}}^{ + }$ ions in the interlayers [53]. The single-connected oxygen atoms in the layers can induce strong interactions with ${\text{NH}}_{{4}}^{ + }$ ions. With the loss of ${\text{NH}}_{{4}}^{ + }$ ions, the weakened interaction and enlarged repulsive force can lead to an expanded interlayer spacing. The phase transition from NH_4_V_4_O_10_ to V_2_O_5_ is attributed to the extraction of cation ${\text{NH}}_{{4}}^{ + }$ completely at a higher temperature, leading to rearrangement of the bi-layered structure. The layer-structured V_2_O_5_ is formed by stacking the single layer of distorted VO_5_ square pyramids shared corners or edges by van der Waals interactions [55].

The Raman spectra of NH_4_V_4_O_10_ and NVO in Fig. 1c are approximate to the character of vanadium pentoxide, indicating the similar VO framework with vanadium pentoxide [47]. The similar spectra suggest that the structure has not much change after heat treatment. The peak at 147 cm^−1^ in NVO is attributed to the bending vibration of –V–O–V–O– chains and exhibits a long-range order [54]. The V = O bending mode appears at 280 cm^−1^and a red shift in NVO means the elongation of V = O along the c-direction, which is consistent with the expanded interplanar spacing [21]. The bands at 408 and 508 cm^−1^ could be assigned to V–O–V banding and stretching mode, respectively [56]. The peaks located at 147, 408, and 508 cm^−1^ take blue shift compared to NH_4_V_4_O_10_ suggests the strong interaction between V and O because of the higher oxidation state of V after heat treatment. The band at 694 cm^−1^ is owing to the coordination of vanadium atoms with three oxygen atoms [57]. The band at 991 cm^−1^ is assigned to V = O stretching of distorted octahedra. In the FTIR spectra (Fig. 1d), the peak at 496 cm^−1^ originates from the bending vibration of the V–O bonds. The band at 750 cm^−1^ is due to antisymmetric stretches of the V–O–V bridges from V^4+^ and V^5+^ ions. The peak becomes strong in NVO, indicating the V–O bond becomes strong because a fraction of V^4+^ is oxidized to V^5+^ [58]. The absorption centered around 996 cm^−1^ is assigned to the stretching mode of V = O. The distorted octahedral structure in NH_4_V_4_O_10_ results in the splitting band of V = O stretching. The merge of bands takes places due to the loss of ammonium cations in NVO. The peaks at around 1412 and 3168 cm^−1^ in NH_4_V_4_O_10_ correspond to the bending and stretching modes of N–H, respectively, confirming the presence of ${\text{NH}}_{{4}}^{ + }$ [59]. The peak intensity became weak in NVO because of few ${\text{NH}}_{{4}}^{ + }$ ions between vanadium oxide layers [60].

**Fig. 2:**
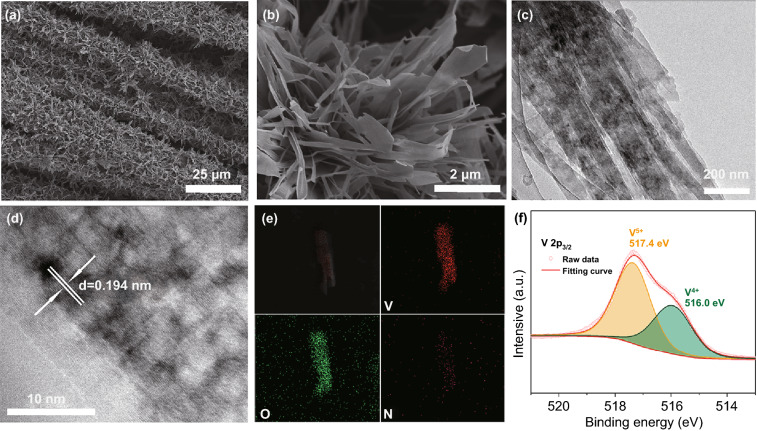
Structural and morphological characterization of the NVO nanosheets. **a, b** SEM images, the ultrathin NVO sheets are grown on the carbon cloth uniformly. **c** TEM and **d** HRTEM images, a lattice spacing of 0.194 nm corresponds to the spacing of (403) plane. **e** Elemental mappings of N, O, and V. Few N elements are detected due to the loss of ammonium ions. **f** XPS spectrum of V 2p_3/2_

SEM images in Fig. 2a, b show that the ultrathin NVO nanosheets are grown on the activated carbon cloth uniformly. The hydrophilic surface of activated carbon cloth is suitable for the uniform growth of nanosheets (Fig. S2). The nanosheets almost maintain the original morphology after annealing in 300 °C compared to the pristine NH_4_V_4_O_10_ (Fig. S3). TEM images (Fig. 2c, d) display the ultrathin nanosheets with a lattice spacing of 0.194 nm, which corresponds to the spacing of (403) plane of NH_4_V_4_O_10_. EDS mapping in Fig. 2e reveals the homogeneous distribution of N, V, and O elements in NVO nanosheets. The trace amount of N element suggests the loss of many ammonium ions between the layers. The high resolution XPS spectrum is used to analyze the valence state of V in two electrodes shown in Figs. 2f and S3. For NVO, the peaks at 517.4 ad 516.0 eV are assigned to the V 2p_3/2_ electrons of V^5+^ and V^4+^, respectively [61, 62]. The ratio of V^5+^/V^4+^ can be calculated by the integration areas of the fitted peak areas. The V^5+^ ratio of 60.4% in NVO is higher than that of NH_4_V_4_O_10_ (51.6%) because of the oxidation of V^4+^ after the heat treatment in air.

### Electrochemical Reaction Kinetics

**Fig. 3:**
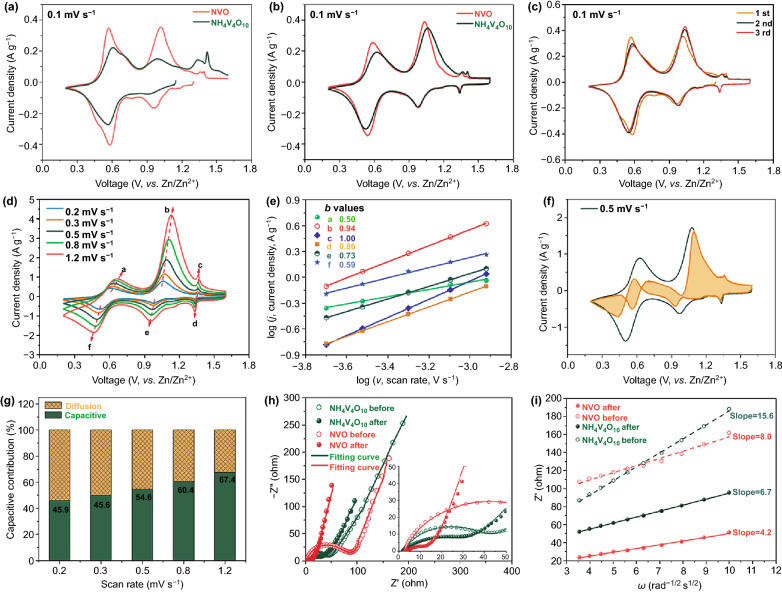
Electrochemical reaction kinetics. Comparison of **a** 1^st^ cycle and **b** 3^rd^ cycle CV curves of NH_4_V_4_O_10_ and NVO at a scan rate of 0.1 mV s^−1^. **c** CV curves of NVO electrode in the first three cycles, the overlapped curves mean a highly reversible reaction. **d** CV curves of NVO at different scan rates. **e** The relationship between peak currents and scan rates. **f** Capacitive contribution at 0.5 mV s^−1^ in NVO (54.6%). **g** The percentages of capacitive and diffusion contributions at different scan rates, increasing from 45.9% to 67.4%. **h** Nyquist plots of NH_4_V_4_O_10_ and NVO before and after CV test. **i** Relationship between the real part of impedance and low frequencies, smaller slopes of the lines mean fast zinc ion diffusion

The first cycle of CV curves at 0.1 mV s^−1^ of NH_4_V_4_O_10_ and NVO is shown in Fig. 3a, and these multiple redox peaks originate from the inequivalent active sites resulting from the inequivalent vanadium cations, leading to a multistep insertion/extraction of zinc ions into interlayer spacing between layers or intralayer spacing in the layers [63]. The first discharge peak of NVO is stronger than that of NH_4_V_4_O_10_, which is possibly due to the high content of V^5+^ and few ammonium ions. Compared to NH_4_V_4_O_10_, more V^5+^ ions are reduced to V^4+^ and the zinc ions are easier to insert into interlayer at high voltage in NVO, providing more capacity. The strong oxide peak of NH_4_V_4_O_10_ at high voltage is attribute to the result of deammoniation. Comparing the third cycle at 0.1 mV s^−1^ in Fig. 3b, both NVO and NH_4_V_4_O_10_ have two main pairs of redox peaks at around 1.0 and 0.5 V, corresponding to the V^5+^/V^4+^ and V^4+^/V^3+^ redox pairs, respectively, and a pair of small peaks at about 1.3 V is also observed, which could be assigned to the occupancy in the intralayer spacing of VO polyhedra [61]. The first cycle of CV curves between NVO and NH_4_V_4_O_10_ is quite different due to the irreversible deammoniation in NH_4_V_4_O_10_, while the similar CV curves in third cycle are associated with the reduced deammoniation. The voltage gaps of redox peaks are summarized in Table 1. The NVO electrode shows smaller voltage gaps of both V^5+^/V^4+^ and V^4+^/V^3+^ redox pairs than those of NH_4_V_4_O_10_ in the first and third cycle of CV, respectively, suggesting the rapid ion diffusion and fast redox reaction kinetics. Both NH_4_V_4_O_10_ and NVO in third cycle display increased voltage gaps for V^4+^/V^3+^ compared to the first cycle which are possibly ascribed to the incomplete deintercalation of zinc ions [64]. Figure 3c shows the CV curves of NVO electrode in the first three cycles. The overlapped CV curves mean a highly reversible reaction of the NVO cathode without the irreversible deammoniation in the ZIBs. It’s worth noting that the discharge peak around 0.6 V shifts to a lower voltage in the subsequent cycles while other peaks move to higher voltage. Such changes also appear in NH_4_V_4_O_10_, which are attributed to the distorted VO polyhedra resulting from the residual Zn^2+^ in the layers as discussed before. The differently curved shapes of the first two cycles in NH_4_V_4_O_10_ imply an irreversible reaction (Fig. S5) because many ${\text{NH}}_{{4}}^{ + }$ groups between the layers are expelled from the open spaces during the intercalation of zinc ions. The CV curves of V_2_O_5_ is quite different from NVO and NH_4_V_4_O_10_ due to the different layered structure for the Zn^2+^ insertion/extraction (Fig. S7a).

**Table 1:** Comparison of the peak positions and voltage gaps of three samples in the first and third cycle of CV

Sample		Peak position in the first cycle(V)	Voltage gap (V)	Peak position in the third cycle(V)	Voltage gap (V)
NVO	V^5+^/V^4+^	1.01/0.96	0.05	1.03/0.98	0.05
	V^4+^/V^3+^	0.57/0.58	0.01	0.59/0.54	0.05
NH_4_V_4_O_10_	V^5+^/V^4+^	0.99/0.90	0.09	1.06/0.98	0.08
	V^4+^/V^3+^	0.61/0.56	0.05	0.62/0.53	0.09

The CV curves at different scan rates from 0.2 to 1.2 mV s^−1^ were measured to analyze the electrochemical reaction kinetics. All CV curves show similar shapes with the increase of scan rate as shown in Fig. 3d. The cathodic peaks shift toward higher potential and the anodic peaks move to lower voltages because of the polarization effect. The relationship of peak currents (*i*) and sweep rate (*v*) was investigated by using the following equation [65, 66]:

1 $$i = av^{b}$$

where *a* and *b* are adjustable parameters. The value of *b* can be determined by the slope of the straight line of log *i* vs log *v*. A *b*-value of 1 indicates that the charge storage is surface-capacitive dominated, while the *b*-value of 0.5 represents a mass diffusion-controlled process. The *b*-values of peaks a-f in NVO were calculated to be 0.50, 0.94, 1.0, 0.86, 0.73, 0.59, respectively (Fig. 3e), whereas the corresponding values in NH_4_V_4_O_10_ were 0.50, 0.9, 1.0, 0.76, 0.65, 0.50 (Fig. S6). The *b*-values suggest that the kinetics of the NVO and NH_4_V_4_O_10_ are controlled by a combination of diffusion and capacitive behaviors, and the higher *b*-values in NVO indicate faster ion diffusion.

In addition, the contributions of capacitive (*k*_*1*_*v*) and diffusion-controlled (*k*_*2*_*v*^*1/2*^) processes could be quantitatively calculated by the current density (*i*) at a particular potential (V) and scan rate (*v*), based on the following equation [67, 68]:

2 $$i(V) = k_{1} v + k_{2} v^{1/2}$$

The fitted CV curve at a scan rate of 0.5 mV s^−1^ is shown in Fig. 3f, in which the shadow area represents the capacitive contribution with a high value of 54.6%. With increasing scan rates from 0.2 to 1.2 mV s^−1^, the contribution ratio of capacitive increases from 45.9% to 67.4% (Fig. 3g), indicating that the electrochemical behavior of the NVO nanosheets is mainly dominated by the capacitive process.

Nyquist plots consist of a semi-circle in the high-frequency section and a straight line in the low-frequency region. The diameter of the semicircle represents the charge transfer resistance (*R*_ct_) and the slope of the line represents Warburg resistance (Z_W_) associating with ion diffusion. In Fig. 3h, the *R*_ct_ of NVO electrode before CV test is about 81 Ω, which is higher than the 42 Ω of NH_4_V_4_O_10_. However, after 15 cycles of CV test, electrodes are activated, leading to reduced *R*_ct_ of two samples, the *R*_ct_ of NVO is decreased to 11 Ω, which is lower than the 30 Ω of NH_4_V_4_O_10_, due to fewer ammonium ions and larger interlayer spacing than NH_4_V_4_O_10_. The relationship between low frequencies and the real part of impedance can be used to calculate the Zn-ion diffusion coefficients ($D_{{Zn^{2 + } }}$) (details shown in ESI) [69, 70]. The NVO electrode exhibits a higher $D_{{Zn^{2 + } }}$ of 2.4 × 10^−13^ cm^2^ s^−1^ than NH_4_V_4_O_10_ of 6.3 × 10^−14^ cm^2^ s^−1^, and retains the superiority after 15 cycles of CV test, with 8.7 × 10^−13^ cm^2^ s^−1^ higher than NH_4_V_4_O_10_ of 3.4 × 10^−13^ cm^2^ s^−1^. These results show that the NVO electrode presents small charge transfer resistance and high Zn-ion diffusion coefficient due to the weak interaction force between zinc ions and ammonium ions and large interlayer spacing, which are favorable for fast redox reaction.

### Electrochemical Properties of NVO

**Fig. 4:**
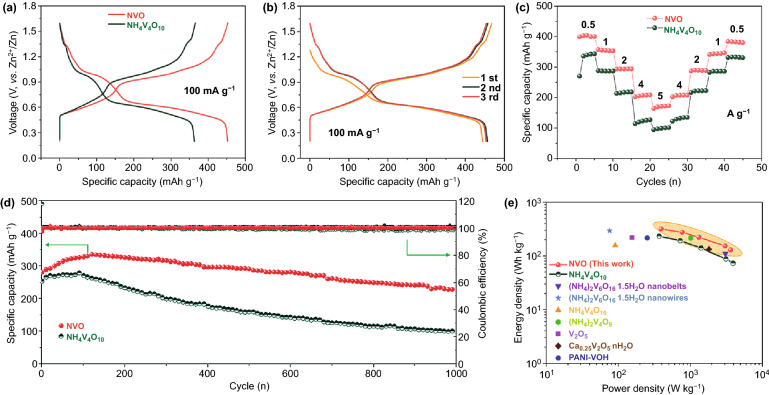
Electrochemical performance of NVO and NH_4_V_4_O_10_. **a** Comparison of GCD plots at 100 mA g^−1^. **b** GCD curves of NVO in first three cycles. **c** Rate performance of NVO and NH_4_V_4_O_10_. **d** Cycling stability with the corresponding coulombic efficiencies at 2 A g^−1^. **e** Ragone plots in comparison with other cathodes, such as (NH_4_)_2_V_6_O_16_·1.5H_2_O nanobelts [48], (NH_4_)_2_V_6_O_16_·1.5H_2_O nanowires [32], NH_4_V_4_O_10_ [50], (NH_4_)_2_V_4_O_9_ [51], V_2_O_5_ [73], Ca_0.25_V_2_O_5_·nH_2_O [74], PANI-VOH [75]

The GCD profiles of NVO and NH_4_V_4_O_10_ at a current density of 100 mA g^−1^ are compared in Fig. 4a. The plateaus during the charge/discharge process are consistent with the CV results. Obviously, the process of Zn^2+^ intercalation can be divided into two steps in NVO and NH_4_V_4_O_10_. In the first step, the reduction of V^5+^ to V^4+^ in NVO delivers a capacity of ∼175 mAh g^−1^, which is higher than that of NH_4_V_4_O_10_ (∼120 mAh g^−1^) due to the higher percentage of V^5+^ and large interlayer spacing for ions insertion. In the second step, the V^4+^ deriving from the first step and intrinsic V^4+^ ions are reduced to V^3+^, delivering a capacity of ∼280 mAh g^−1^. In addition, a lower charge plateau and a higher discharge plateau are observed in NVO electrode compared to NH_4_V_4_O_10_ electrode, indicating that the NVO electrode possess higher energy efficiency than NH_4_V_4_O_10_ electrode. The first three voltage profiles of NVO are shown in Fig. 4b, and the delivered specific capacity is 457 mAh g^−1^ at 100 mA g^−1^, which is high than that of other ammonium vanadate cathodes at the same current density, such as (NH_4_)_0.5_V_2_O_5_ (418.4 mAh g^−1^) [49], (NH_4_)_2_V_4_O_9_ (378 mAh g^−1^) [51], (NH_4_)_2_V_6_O_16_·1.5H_2_O (385 mAh g^−1^) [32], and NH_4_V_3_O_8_·0.5H_2_O (423 mAh g^−1^) [71]. The initial coulombic efficiency of NVO could reach up to 97%, much higher than that of NH_4_V_4_O_10_ (85%, Fig. S7), because a small number of ${\text{NH}}_{{4}}^{ + }$ ions in the interlayers do not act as barriers for the first intercalation of zinc ions. In the subsequent two cycles, the specific capacities are slightly increased due to the activated process and the GCD curves overlap well, suggesting a good reversibility of the NVO electrode during electrochemical reactions. However, the voltage profiles of NH_4_V_4_O_10_ electrode exhibit different sharps in the first three cycles due to the irreversible deammoniation (Fig. S7). Figure 4c shows the rate performance of NVO and NH_4_V_4_O_10_. The NVO electrode delivers an initial specific capacity of 397 mAh g^−1^ at a current density of 0.5 A g^−1^, and the capacity of NH_4_V_4_O_10_ electrode is 270 mAh g^−1^. For the second cycle, the capacity of NVO could reach to 403 mAh g^−1^, which is higher than that of NH_4_V_4_O_10_ (336 mAh g^−1^). The specific capacities of NVO at different current densities of 1, 2, 4, and 5 A g^−1^ are 357, 294, 203, and 170 mAh g^−1^, respectively, while NH_4_V_4_O_10_ delivers lower capacities of 288, 214, 114, and 95 mAh g^−1^ at the corresponding current densities. When the current densities decrease back to 0.5 A g^−1^, the capacities recover to the initial values, suggesting a stable crystal structure and great electrochemical reversibility. Compared to NH_4_V_4_O_10_ and V_2_O_5_, the improved capacity and rate performance of NVO could be attributed to the large interlayer spacing and abundant active sites for fast zinc ion diffusion and facile accommodation (Fig. S7). The ordinary rate performance of free-standing NVO could be attributed to no additional conductive agent, which restricts the charge transfer. The galvanostatic charge–discharge profiles of NVO at different current densities are shown in Fig. S8. Cycling stability and corresponding Coulombic efficiencies of NVO and NH_4_V_4_O_10_ electrodes were evaluated at a current density of 2 A g^−1^ in Fig. 4d. The NH_4_V_4_O_10_ electrode remained only ~ 40% retention of the initial capacity after 1000 cycles and exhibited fluctuant Coulombic efficiencies during cycling test. The fast capacity fading could be attributed to structure degradation caused by the irreversible deammoniation during the long-term cycle. For NVO electrode, the first discharge capacity was 280 mAh g^−1^ and reached up to 335 mAh g^−1^ after the initial 120 cycles, due to the electrochemical activation, still maintaining a capacity of 227 mAh g^−1^ after 1000 cycles. The activated process is possibly attributed to the incomplete deintercalation of zinc ions, which act as “pillars” in the interlayer to improve the structural stability and contribute to the enhanced capacity. The microstructure of NVO nanosheets is maintained well after cycling test as shown in Fig. S9. The decay of the specific capacity could be attributed to the aggregation of by-product on the surface of NVO nanosheets during the repeated charge/discharge test. The by-product could be confirmed as Zn_3_V_2_O_7_(OH)_2_·2H_2_O (JCPDS No. 87–0417) from the XRD of sample after cycling, which has a side effect on the cycling performance of NVO (Fig. S11) [72]. Besides, the NVO showed high Coulombic efficiencies of near to 100% in the 1000 cycles, indicating the high reversibility. The improved cycling stability could be attributed to the little change of the structure without the side effect of deammoniation and the residual ammonium ions after heat treatment stabilizing the structure. The comparison of electrochemical performance of previous reported ammonium vanadates and our NVO nanosheets is given in Table S1. The Ragone plots (Fig. 4e) comparing NVO and NH_4_V_4_O_10_ nanosheets with some typical cathodes such as (NH_4_)_2_V_6_O_16_·1.5H_2_O nanobelts [48], (NH_4_)_2_V_6_O_16_·1.5H_2_O nanowires [32], NH_4_V_4_O_10_ [50], (NH_4_)_2_V_4_O_9_ [51], V_2_O_5_ [73], Ca_0.25_V_2_O_5_·nH_2_O [74], PANI-VOH [75]. A remarkable energy density of 317 Wh kg^−1^ was delivered at a power density of 392 W kg^−1^ based on the mass of active materials, which was higher than that of NH_4_V_4_O_10_ (232.5 Wh kg^−1^ at 367 W kg^−1^).

### Charge Storage Mechanism of NVO

**Fig. 5:**
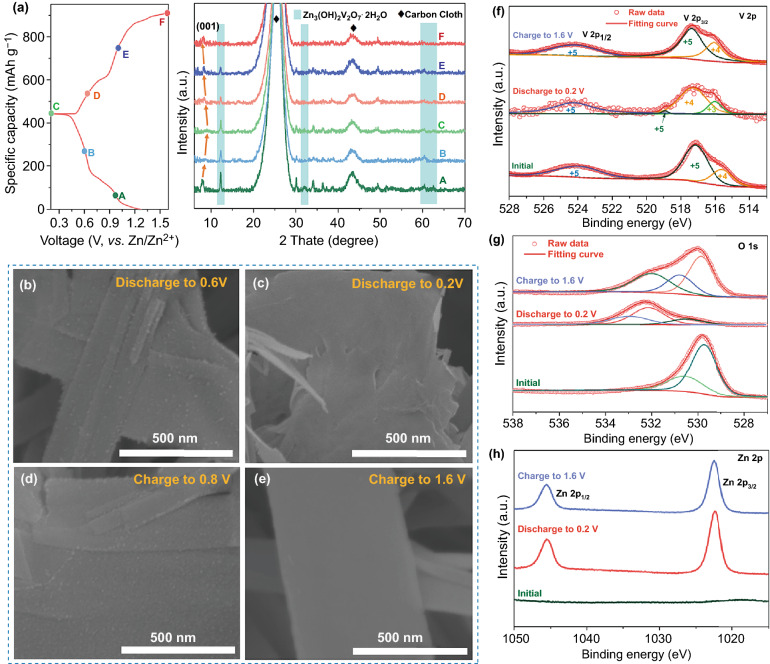
Electrochemical reaction process studies. **a** Ex situ XRD patterns of NVO in different discharge/charge states, the shifts and recovery of (001) peaks suggest a reversible reaction in the electrochemical processes. Ex situ SEM of NVO in the first discharge/charge process, **b** discharge to 0.6 V, **c** discharge to 0.2 V, **d** charge to 0.8 V, **e** charge to 1.6 V. Ex situ XPS spectra of **f** V 2p, **g** O 1 s, **h** Zn 2p in the initial, discharge to 0.2 V and charge to 1.6 V states in the first cycle

Some *ex-situ* characterizations were employed to study the charge storage mechanism of NVO electrode in Fig. 5. The phases changes of NVO nanosheets during the first charging-discharging process were analyzed using ex situ XRD characteristics in Fig. 5a. Compared to pristine NVO, new peaks located at 12.3°, 32.1°, 60.6°, and 62.6° are indexed to Zn_3_V_2_O_7_(OH)_2_·2H_2_O (JCPDS No. 87–0417), which has been reported in previous studies [47, 52, 76]. Generally, Zn^2+^ could be coordinated with water molecules in the aqueous electrolyte to form large [Zn(H_2_O)_6_]^2+^ [77]. Because of the weakened O–H bond affected by Zn^2+^ in H_2_O, the OH^−^ might originate from the broken O–H bond and react with VO in the layers to form Zn_3_V_2_O_7_(OH)_2_·2H_2_O. At the same time, we suspect that the generating H^+^ would not exist in the electrolyte but possibly insert into the cathode materials [78]. In addition, during the first discharge, the (001) peak of NVO shifted slightly to high angle area, indicating shrinkage of the interlayer spacing upon Zn-intercalation. When discharged to 0.2 V, the (001) peak moves to the high angle of 9.06° with the interlayer distance decreased to 9.8 Å, which is possibly attributed to the strong electrostatic interaction between Zn^2+^ and negative single-connected oxygen. Upon the first charge, the (001) peak of NVO returns and the peaks of Zn_3_V_2_O_7_(OH)_2_·2H_2_O disappear, demonstrating the reversibility of the phase transition.

Ex situ SEM was conducted to study the changes of morphologies during the first charge/discharge process in Fig. 5b-e. When discharged to 0.6 V, some nanoparticles appear on the surface of the NVO nanosheets (Fig. 5b), which could be the growth of Zn_3_V_2_O_7_(OH)_2_·2H_2_O. After fully discharged, the surface of the electrode is fully covered by this by-product (Fig. 5c). When charged to 0.8 V, the Zn_3_V_2_O_7_(OH)_2_·2H_2_O layer begins to be decomposed and form the nanoparticles (Fig. 5d). As shown in Fig. 5e, after the electrode is charged to 1.6 V, the Zn_3_V_2_O_7_(OH)_2_·2H_2_O by-product is decomposed completely and the surface becomes smooth. The results of ex situ SEM suggest that the formation and decomposition of Zn_3_V_2_O_7_(OH)_2_·2H_2_O are highly reversible. This process was further confirmed by the ex situ TEM in Fig. S12. A lattice spacing of 0.246 nm can be observed at the fully discharged state, corresponding to the (201) plane of the new phase of Zn_3_V_2_O_7_(OH)_2_·2H_2_O (Fig. S12a). After charging to 1.6 V, the electrode exhibits the characteristic of NVO, suggesting the good reversibility (Fig. S12b). EDS mapping in Fig. S12c, d shows the distribution of N, V, O, and Zn elements at the fully discharged and charged states. The Zn signal at the fully charged could originate from the absorbed Zn^2+^ or Zn^2+^ existing in the crystal lattice.

The V 2p XPS spectra of NVO at different electrochemical stages are shown in Fig. 5f. When discharged to 0.2 V, the V^5+^ cations are reduced to V^4+^ and V^3+^ due to the insertion of zinc ions. The existence of V^5+^ state could be attributed to the Zn_3_V_2_O_7_(OH)_2_·2H_2_O by-product and the incomplete redox reaction results that the delivered specific capacity is lower than theoretical capacity. In addition, the peaks shift towards higher binding energy in the fully discharged state because the inserted zinc ions could affect the distribution of electron [79]. After charged to 1.6 V, the V cations are oxidized, and the V 2p spectrum is similar to that of initial state, suggesting good reversible redox reactions. Figure 5g shows the XPS spectra of O 1 s. After the first discharging process, the peaks located at around 532.5 and 533.0 eV are attributed to water molecules and OH^−^, respectively, indicating to the insertion of hydrated zinc ions and the formation of Zn_3_V_2_O_7_(OH)_2_·2H_2_O [80]. After charging to 1.6 V, the H_2_O signal is retained while the OH^−^ signal disappears, which further suggests the decomposition of Zn_3_V_2_O_7_(OH)_2_·2H_2_O. There is no peaks of Zn 2p in the pristine NVO (Fig. 5h), while two peaks corresponding to Zn 2p_1/2_ (1045.6 eV) and Zn 2p_3/2_ (1022.5 eV) appear in the fully discharged state, indicating the intercalation of zinc ions [81]. The Zn 2p signals in the fully charged state are also detected, which is consistent with the results of ex situ TEM.

**Fig. 6:**
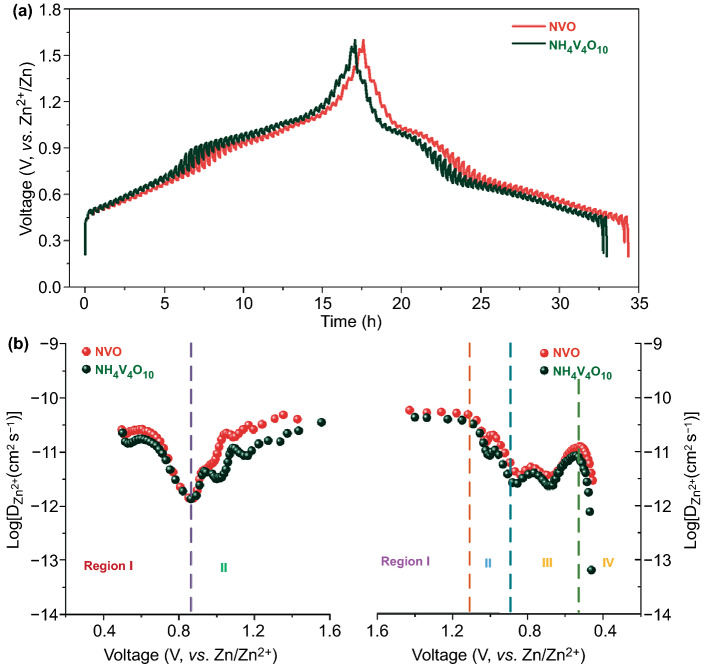
Analysis of ions insertion/extraction process. **a** GITT tests at a current density of 50 mA h g^−1^. **b** Corresponding ion diffusion coefficients of NVO and NH_4_V_4_O_10_

GITT plots were measured at a current density of 50 mA g^−1^ to calculate the diffusion coefficients of the zinc ions as shown in Fig. 6a, b (details supplied in Supporting information) [82, 83]. The *D*_Zn_ values of NVO during both the insertion and extraction processes are within the orders of 10^−10^ to 10^−12^ cm^2^ s^−1^, superior to those of NH_4_V_4_O_10_, indicating the fast Zn-ion diffusion ability, which is agreement with the EIS results. From ex situ characterizations and GITT plots, the zinc ions insertion/extraction process in NVO could be deduced. In the discharging process, the *D*_Zn_ value goes through four stages: stabilization, gradual decline, fluctuating rise, and sharp decline. The *D*_Zn_ values remain unchanged at the beginning of discharging process (Region I) due to large interlayer space for the insertion of hydrated zinc ions. When discharged to 0.9 V (Region II), the *D*_Zn_ values decrease gradually which is possibly attributed to the difficult insertion of large sized [Zn(H_2_O)_6_]^2+^. Then, the *D*_Zn_ value exhibits a tendency of fluctuating increasement (Region III) which is possibly attributed to the desolvation of [Zn(H_2_O)_6_]^2+^ and easier intercalation process of small Zn^2+^. After discharged to 0.5 V (Region IV), the *D*_Zn_ value decreases dramatically because no channels are available for ions intercalation. When charged to 0.8 V (Region I), the *D*_Zn_ value decreases gradually owing to the deintercalation of Zn^2+^. After 0.8 V (Region II), the increase in *D*_Zn_ value is attributed to decomposition of Zn_3_V_2_O_7_(OH)_2_·2H_2_O and then producing more Zn^2+^.

## Conclusions

Pre-removing some ammonium ions from NH_4_V_4_O_10_ not only expanded the interlayer spacing but also produced many active sites, which offered more diffusion pathways, facilitated the zinc ions insertion/extraction and alleviate cycling decay caused by the irreversible dissolution of ammonium ions during cycles. The NVO electrode exhibits a high initial Coulombic efficiency of 97% compared to 85% of NH_4_V_4_O_10_ electrode and delivers a high specific capacity of 457 mAh g^−1^ at a current density of 100 mA g^−1^ compared to 363 mAh g^−1^ of NH_4_V_4_O_10_ electrode as well as a long-term cycling stability (81% of initial capacity after 1000 cycles) compared to 40% of NH_4_V_4_O_10_ electrode. The *ex-situ* characterizations (XRD, SEM, TEM, and XPS) demonstrated reversible Zn_3_V_2_O_7_(OH)_2_·2H_2_O formation/decomposition in NVO during charge/discharge processes. This work provides a novel strategy of deionized method for designing high-performance cathode materials for ZIBs and other multivalent ion batteries.

## Supplementary Information

Below is the link to the electronic supplementary material.

Supplementary file1 (PDF 1210 kb)
