# Supplementary material for: Enhanced Reversible Zinc Ion Intercalation in Deficient Ammonium Vanadate for High-Performance Aqueous Zinc-Ion Battery
Source: Nanomicro Lett. 2021 Apr 30;13:116. doi: 10.1007/s40820-021-00641-3 (PMC8087727; doi:10.1007/s40820-021-00641-3)
Supplement: Supplementary file 1 — Supplementary file1 (PDF 1210 kb) [file 40820_2021_641_MOESM1_ESM.pdf]

Supporting Information for

Enhanced Reversible Zinc Ion Intercalation in Deficient Ammonium Vanadate for High-Performance Aqueous Zinc-Ion Battery

Quan Zong[1, 2], Wei Du[2], Chaofeng Liu[1], Hui Yang[2], Qilong Zhang[2, *], Zheng Zhou[2], Muhammad Atif[3, 4], Mohamad Alsalhi[3, 4], Guozhong Cao[1, *]

[1]Department of Materials Science and Engineering, University of Washington, Seattle, WA, 98195, USA

[2]School of Materials Science and Engineering, State Key Lab Silicon Mat, Zhejiang University, Hangzhou 310027, P. R. China

[3]Research Chair on Laser Diagnosis of Cancers, Department of Physics and Astronomy, College of Science, King Saud University, 11451, Riyadh, Saudi Arabia

[4]Department of Physics and Astronomy, College of Science, King Saud University, 11451, Riyadh, Saudi Arabia

*Corresponding authors. E-mail: gzcao@uw.edu (G. Cao), mse237@zju.edu.cn (Q. Zhang)

Supplementary Tables and Figures

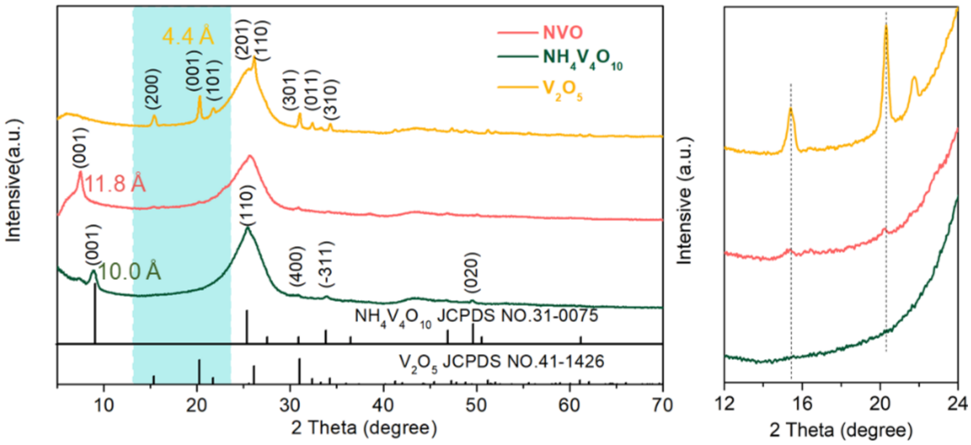

Fig. S1 XRD patterns of NVO, $NH_4V_4O_{10}$ and V_2O_5 and the enlarged area (12°~24°). The V_2O_5 sample is obtained via heat treatment at 400 °C. Some new weak peaks assigned to V_2O_5 can be observed in NVO as shown in the enlarged patterns, which can be attributed to uneven heat transfer.

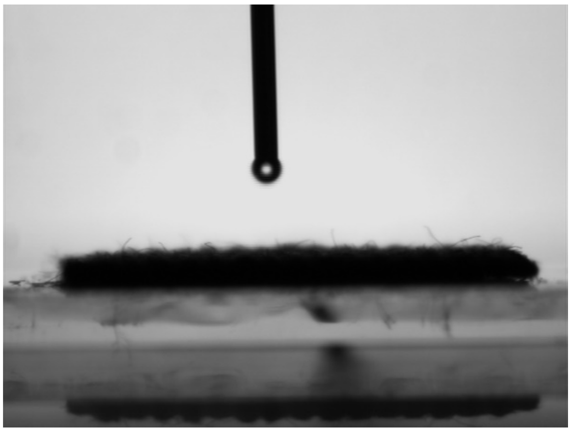

Fig. S2 Contact angle of carbon cloth after treatment. The hydrophilic surface of activated carbon cloth is suitable for the uniform growth of nanosheets

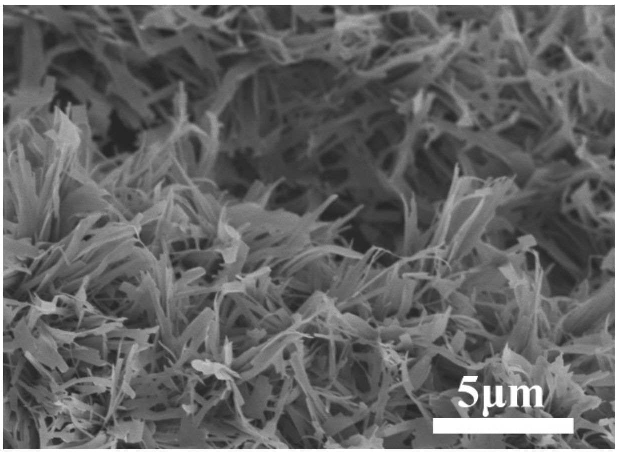

Fig. S3 SEM images of $NH_4V_4O_{10}$ nanosheets

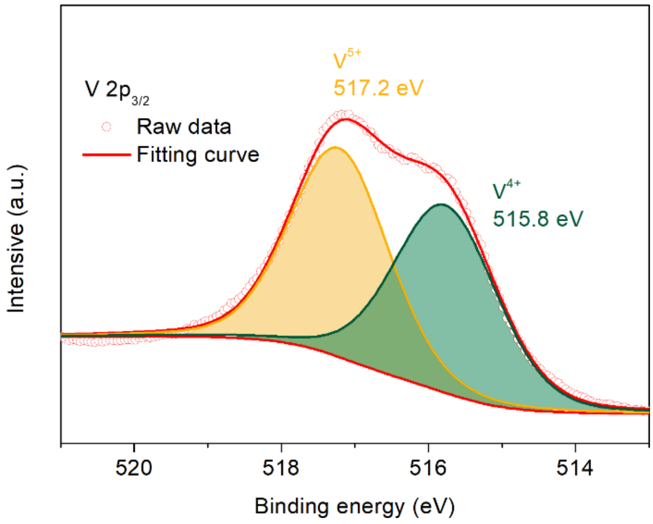

Fig. S4 XPS spectrum of V $2p_{3/2}$ of $NH_4V_4O_{10}$

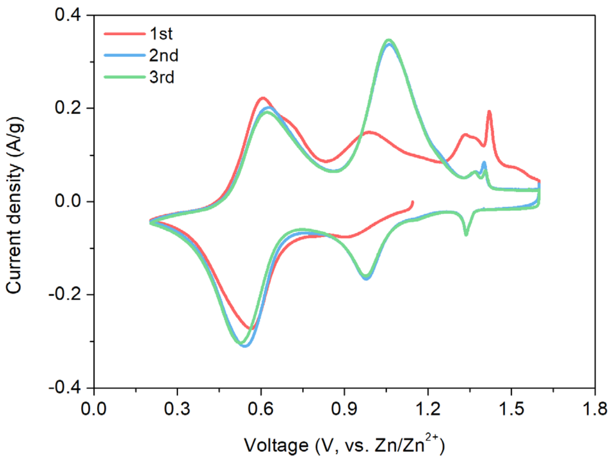

Fig. S5 CV curves of $NH_4V_4O_{10}$ nanosheets in the first three cycles. There is an irreversible reaction process in the first two cycles

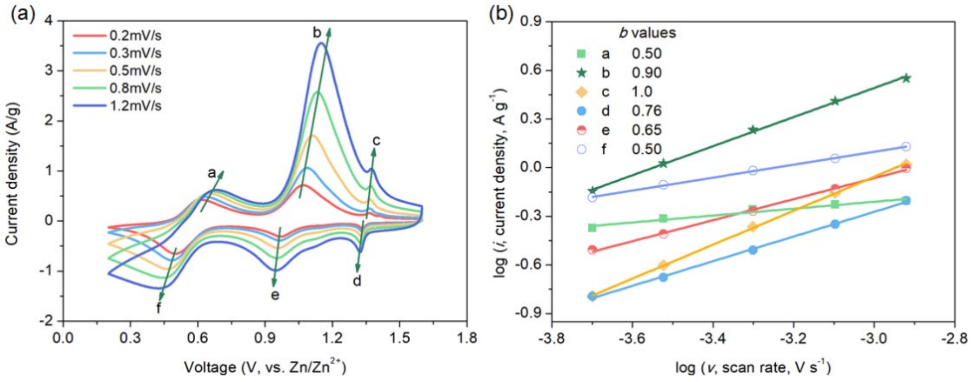

Fig. S6 (**a**) CV curves of $NH_4V_4O_{10}$ at different scan rates. (**b**) The relationship between peak currents and scan rates

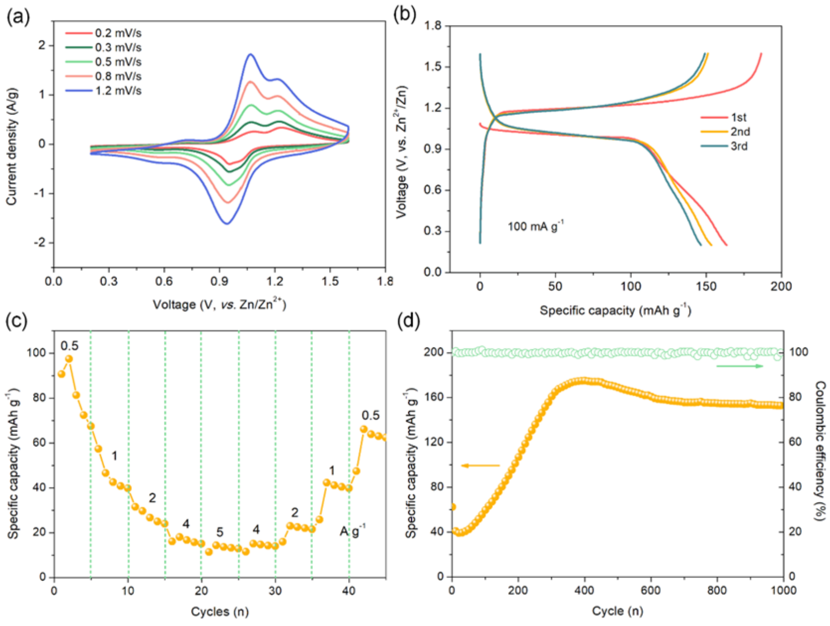

Fig. S7 (**a**) CV curves of V_2O_5 at different scan rates. (**b**) GCD curves of NVO in first three cycles. (**c**) Rate performance. (**d**) Cycling performance

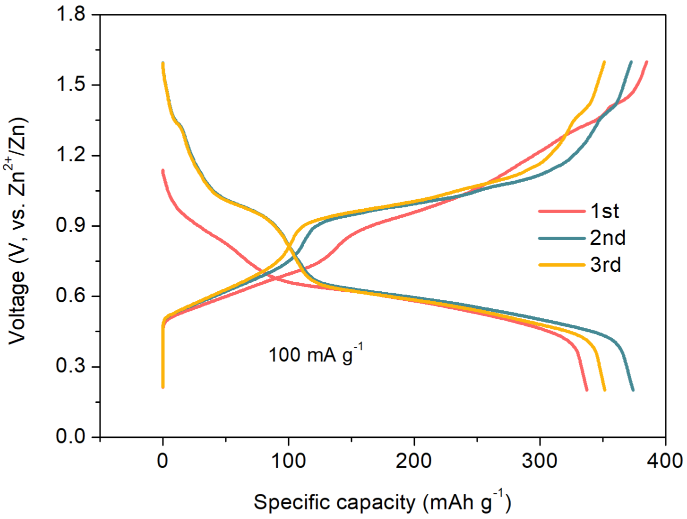

Fig. S8 GCD curves of $NH_4V_4O_{10}$ nanosheets in first three cycles at 100 mA g^{-1}. The $NH_4V_4O_{10}$ nanosheets exhibit an irreversible reaction

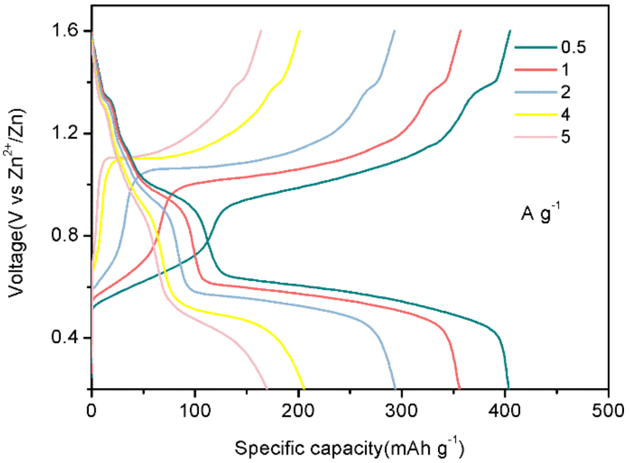

Fig. S9 GCD curves of NVO at different current densities

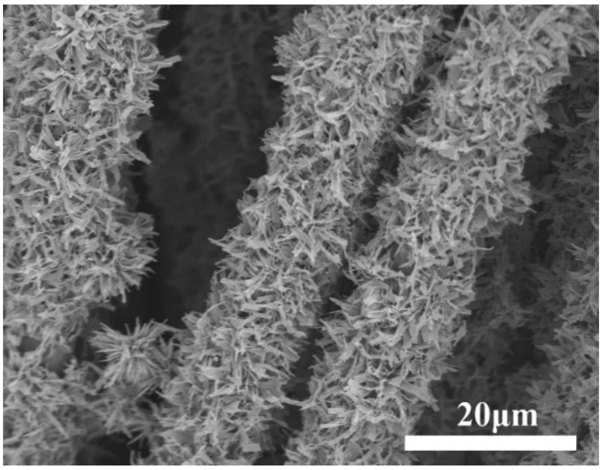

Fig. S10 SEM image of NVO electrode after 1000 cycles

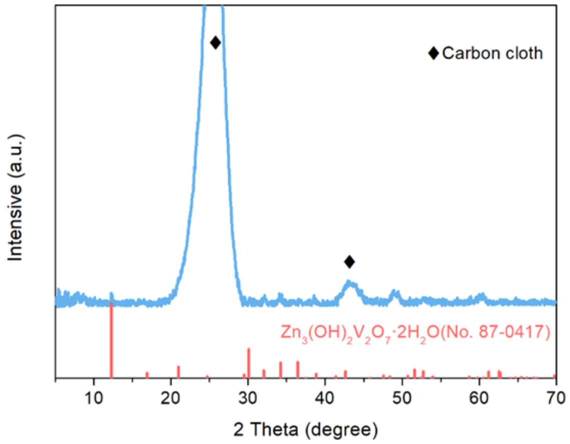

Fig. S11 XRD pattern of NVO after 1000 cycles

Table S1 Comparison of electrochemical properties of previous reported ammonium vanadates and our work

Electrode	Specific capacity	Rate performance	Cycling performance	Refs.
NVO	457 mAh g^{-1} at 0.1 A g^{-1}	170 mAh g^{-1} at 5 A g^{-1}	81% (1000)	This work
$(NH_4)_2V_3O_8/C$	356 mAh g^{-1} at 0.1 A g^{-1}	232 mAh g^{-1} at 0.5 A g^{-1}	50% (2000)	[S1]
$(NH_4)_2V_4O_9$	376 mAh g^{-1} at 0.1 A g^{-1}	259 mAh g^{-1} at 1 A g^{-1}	86.7% (100)	[S2]
$(NH_4)_xV_2O_5{\cdot}nH_2O$	351 mAh g^{-1} at 0.2 A g^{-1}	204 mAh g^{-1} at 4 A g^{-1}	80% (2000)	[S3]
$NH_4V_4O_{10}$	147 mAh g^{-1} at 0.2 A g^{-1}	72 mAh g^{-1} at 2 A g^{-1}	70.3% (5000)	[S4]
$NH_4V_3O_8{\cdot}0.5H_2O$	423 mAh g^{-1} at 0.1 A g^{-1}	266 mAh g^{-1} at 1 A g^{-1}	50.1% (1000)	[S5]

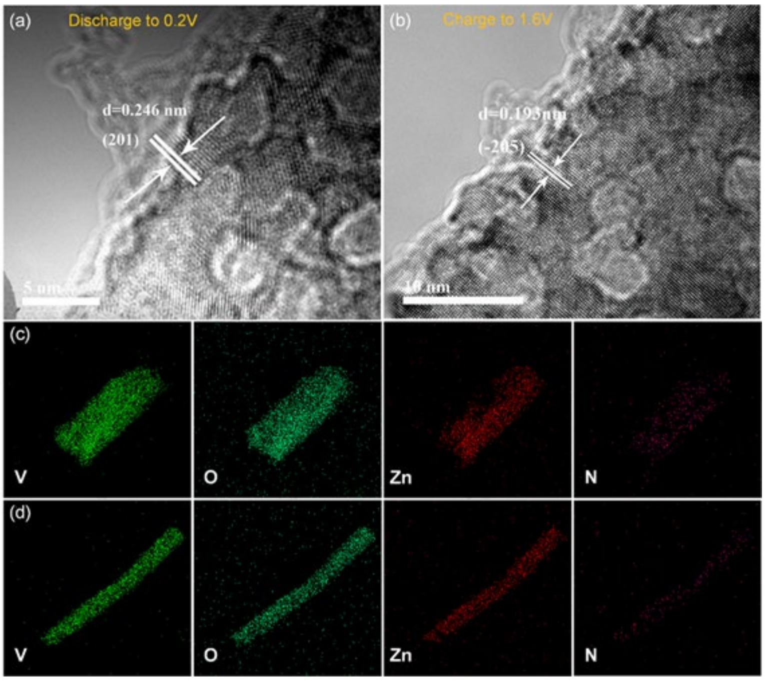

Fig. S12 HRTEM images of the electrode (**a**) discharged to 1.2V and (**b**) charged to 1.6V. EDS mapping in (**c**) the fully discharged state and (**d**) the fully charged state

The Zn-ion diffusion coefficient can be calculated by using the following equation [S6]:

$$D_{Zn^{2+}} = \frac{R^2T^2}{2A^2n^4F^4C^2{\sigma_\omega}^2}$$

where R is the gas constant (8.314 J mol^{-1} K^{-1}), T is the absolute temperature (298 K), A is the surface area of the electrode (1 cm^2), n is number of electrons transferred per molecule (4), F is the Faraday constant(96500 C mol^{-1}), C is concentration of ion (3x10^{-3} mol cm^{-3}) in the electrolyte, D is the diffusion coefficient (cm^2 s^{-1}), and σ_ω is the Warburg coefficient, which is the slope of the fit line for Z' and angular frequency $\omega^{-1/2}$ (equation).

$$Z' = R_D + R_L + \sigma_\omega \omega^{-1/2}$$

The GITT was employed to calculate the ion diffusion coefficient of Zn^{2+} based the following equation [S7, S8]:

$$D_{Zn} = \frac{4}{\pi\tau}\left(\frac{m_B V_M}{M_B A}\right)^2 \left(\frac{\Delta E_s}{\Delta E_\tau}\right)^2$$

where τ is the duration time of the current pulse (600s), m_B is the mass of the active materials, V_M is the molar volume (cm^3 mol^{-1}), M_B is the molecular weight (g mol^{-1}), A is the total contacting area of electrode with electrolyte, and ΔE_s and ΔE_τ are related to the change of steady-state voltage and overall cell voltage for the corresponding step.

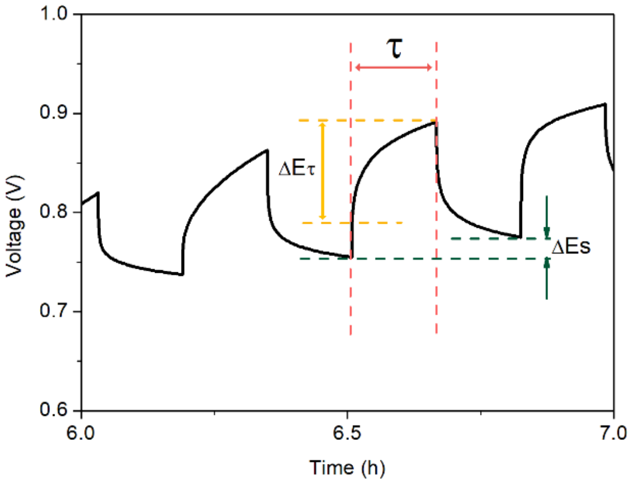

Fig. S13 Definition of ΔEτ, ΔEs and τ

Supplementary References

[S1] H. Jiang, Y. Zhang, L. Xu, Z. Gao, J. Zheng et al., Fabrication of $(NH_4)_2V_3O_8$ nanoparticles encapsulated in amorphous carbon for high capacity electrodes in

aqueous zinc ion batteries. Chem. Eng. J. **382**,122844 (2020). https://doi.org/10.1016/j.cej.2019.122844

[S2] Y. Zhang, H. Jiang, L. Xu, Z. Gao, C, Meng. Ammonium vanadium oxide [$(NH_4)_2V_4O_9$] sheets for high capacity electrodes in aqueous zinc ion batteries. ACS Appl. Energy Mater. **2**(11), 7861-7869 (2019). https://doi.org/10.1021/acsaem.9b01299

[S3] L. Xu, Y. Zhang, J. Zheng, H. Jiang, T. Hu et al., Ammonium ion intercalated hydrated vanadium pentoxide for advanced aqueous rechargeable Zn-ion batteries. Mater. Today Energy **18**, 100509 (2020). https://doi.org/10.1016/j.mtener.2020.100509

[S4] G. Yang, T. Wei, C. Wang, Self-healing lamellar structure boosts highly stable zinc-storage property of bilayered vanadium oxides. ACS Appl. Mater. Interfaces **10**(41), 35079-35089 (2018). https://doi.org/10.1021/acsami.8b10849

[S5] H. Jiang, Y. Zhang, Z. Pan, L. Xu, J. Zheng et al., $NH_4V_3O_8 \cdot 0.5H_2O$ nanobelts with intercalated water molecules as a high performance zinc ion battery cathode. Mater. Chem. Front. **4**(5), 1434-1443 (2020). https://doi.org/10.1039/d0qm00051e

[S6] J. Zheng, C. Liu, M. Tian, X. Jia, E. P. Jahrman et al., Fast and reversible zinc ion intercalation in Al-ion modified hydrated vanadate. Nano Energy **70**, 104519 (2020). https://doi.org/10.1016/j.nanoen.2020.104519

[S7] F. Liu, Z. Chen, G. Fang, Z. Wang, Y. Ca et al., V_2O_5 nanospheres with mixed vanadium valences as high electrochemically active aqueous zinc-ion battery cathode. Nano-Micro Lett. **11**, 25 (2019). https://doi.org/10.1007/s40820-019-0256-2

[S8] X. Chen, L. Wang, H. Li, F. Cheng, J. Chen, Porous V_2O_5 nanofibers as cathode materials for rechargeable aqueous zinc-ion batteries. J. Energy Chem. **38**, 20-25 (2019). https://doi.org/10.1016/j.jechem.2018.12.023
